# The Age of Activewear: Understanding Women’s Casualized Athletic Apparel Habits Through Associations with Psychosocial and Body Image Factors

**DOI:** 10.3390/bs16040586

**Published:** 2026-04-14

**Authors:** Ross C. Hollett, Larissa R. Sharman, Domenic L. D. D’Adamo

**Affiliations:** Psychology and Criminology, Edith Cowan University, Joondalup, WA 6027, Australia; larissa.sharman@hotmail.com (L.R.S.); domdadamo@gmail.com (D.L.D.D.)

**Keywords:** athleisure, body image, objectification, apparel, fashion, marketing, fitspiration

## Abstract

Activewear has become a common component of women’s everyday clothing, yet emerging evidence suggests that exposure to activewear imagery may adversely affect body image. This study aimed to describe women’s activewear engagement across contexts and to investigate how different markers of engagement correspond with positive outcomes such as fitness behavior, body appreciation, and self-esteem, as well as negative outcomes including media pressure, idealized appearance aspirations, and self-objectification. We collected survey data from student (*N* = 455) and community (*N* = 374) samples to assess activewear-related behaviors, including online browsing, social media following, purchasing, and wearing, as well as measures of fitness behaviors, body appreciation, self-esteem, idealized body aspirations, appearance comparisons, perceived media pressure, and self-objectification. Across samples, 40–87% of women engaged with activewear in some form, and 30% reported feeling self-conscious at least half the time they wore it; notably, activewear was worn for exercise less than 50% of the time. Activewear engagement showed positive correlations with fitness behaviors but also with idealized body aspirations, appearance comparisons, media pressure, and self-objectification, while showing no associations with body appreciation or self-esteem. These findings highlight the growing cultural prominence of activewear and suggest that engagement with this clothing trend is linked to both adaptive and risk-related psychological factors, underscoring the need for further research into its broader psychological implications.

## 1. Introduction

The widespread adoption of athletic apparel by women as a form of casual wear has become a global phenomenon (Brice et al., 2022; Perera et al., 2024), evidenced by the immense sales growth across the fashion industry, particularly in developed countries such as the US and Australia (Statistica, 2023; Yahoo Finance, 2023). Estimates suggest that casualized athletic apparel (or “activewear”) global sales will reach USD 450 billion by 2028, with women consistently and substantially outspending men (Statistica, 2025). As the fastest-growing apparel segment among women, there is an urgent imperative to understand the positive and negative impact of activewear on women’s psychological well-being. Specifically, the cultural association between activewear, fitness, diet, convenience, and emphasis of the female physique implicates a range of potential psychosocial and body image benefits and risks (Hollett et al., 2024; Lipson et al., 2020). Importantly, wearing activewear is often marketed by leading brands as a positive influence on women’s mental and physical health by inspiring active lifestyles, creating communities, and enhancing body confidence (Gymshark, n.d.-b; Lorna Jane, n.d.; Ryderwear, n.d.-a; Stax, n.d.). While some research supports links between activewear engagement and a healthy lifestyle orientation (Watts & Chi, 2019), experimental studies show that activewear imagery is threatening to women’s body image and significantly linked to self-objectification, body shame, appearance comparison tendencies, and eating disorder symptoms (Hollett et al., 2024; Hollett & Challis, 2023). These prior findings broadly support the assumptions of both objectification and social comparison theories (Festinger, 1954; Fredrickson & Roberts, 1997), both of which informed the psychosocial perspective adopted in the current study.

As the potential benefits and risks of activewear engagement are still emerging, further exploratory research is necessary. Consequently, the present study had several aims. Firstly, we sought descriptive information on women’s activewear habits by capturing several markers of activewear engagement and wearing preferences across different contexts. Secondly, we explored associations between these activewear engagement markers and positive outcomes, including fitness behaviors, body appreciation, and self-esteem. Thirdly, we explored associations between activewear engagement markers and negative outcomes, including perceived media pressure, idealistic body image aspirations, and self-objectification.

### 1.1. Activewear Preferences and Engagement Habits Amongst Women

Activewear has been described as “clothing designed specifically for fitness and functional movement” (Brice et al., 2022, p. 1) as well as “suitable for sport/gym, though also worn casually” (Hollett & Challis, 2023, p. 386). The rise of activewear as everyday attire reflects broader shifts in consumer culture, which reflect a blend of work and leisure, with evidence suggesting this was accelerated by the COVID-19 pandemic (Australia Post, 2021; Salfino, 2020). Unlike traditional fashion trends, whereby clothing might be segmented by context or activity, modern consumers demand clothing that accommodates multifunctional lifestyles (Crane, 2000; McNeill, 2018; Perera et al., 2024). The proliferation of women-focused activewear brands and their social media presence, particularly post 2019, signals a cultural shift where comfort, visibility, and body-centric feminine aesthetics converge. While this timeframe aligns with pandemic-related changes in how consumers exercised and navigated work–home boundaries, it also illustrates how brands leveraged the transition to remote work to target an emergent niche: consumers seeking comfortable, adaptable clothing to replace the corporate attire they were no longer wearing (Brydges et al., 2021). The present study is valuable because it aimed to highlight whether the perceived cultural benefits of emerging activewear engagement trends are likely to outweigh the risks.

Importantly, modern trends in activewear have increasingly shifted toward form-fitting garments which showcase the female physique whilst offering a range of fabrics and styles appealing to individual preferences (e.g., high/low waisted, seamed/seamless, etc.) (Echt, n.d.; Gymshark, n.d.-b; LSKD, n.d.; Ryderwear, n.d.-b). Notable examples include leggings (or tights), bike shorts, and crop tops. One likely mechanism behind the continued financial growth of activewear has been the successful representation of activewear as a convenient apparel solution for almost every context in which women wear clothing (e.g., fitness venues, shopping centers, social venues, relaxing at home, etc.) (O’Sullivan et al., 2017; Perera et al., 2024; Watts & Chi, 2019). While it is often assumed that activewear has comprehensively transitioned from exercise settings into other contexts, quantitative research is yet to determine to what extent activewear is worn outside of exercise settings. Some preliminary qualitative evidence suggests that women often wear activewear outside of exercise settings and recognize this growing trend among women (Lipson et al., 2020). However, quantitative research is needed to complement these studies by measuring activewear engagement habits among women and exploring associations between these habits and psychosocial factors.

There are several measurable indicators that are useful for understanding women’s relationship with activewear and activewear imagery. Specifically, we suggest the frequency of wearing activewear, the amount spent on activewear, the time spent browsing online for activewear and following activewear brands on social media. Women’s purchasing and wearing habits are critical for understanding women’s investment in activewear, as these behaviors represent a commitment to acquiring activewear and endorsing it during their lifestyle activities. However, few studies have captured purchasing and wearing habits at the individual level. While qualitative evidence suggests that activewear has become a staple part of women’s wardrobes (Brice et al., 2022; Lipson et al., 2020), quantitative estimates of women’s wearing frequency (e.g., in days per week) or estimates of recent spending habits are yet to be reported. Instead, quantitative studies to date have focused on purchase intentions and online browsing habits (Hollett & Challis, 2023; Hollett et al., 2023; Rahulan et al., 2015; Watts & Chi, 2019). Beyond measuring wearing frequency, we also consider it important to capture the contexts in which activewear is worn, as this contextual information can help illuminate patterns of use without directly assessing the motivations behind them.

There are also activewear engagement behaviors related to women’s digital exposure to activewear imagery and marketing, which are valuable to quantify. For instance, online shopping and exposure to activewear imagery on social media are two common digital channels by which women are potentially influenced by activewear (Froehlich, 2022; Griffiths & Stefanovski, 2019; Ryu & Park, 2020). Some preliminary evidence suggests that women browse for activewear online significantly more than most other fashion segments, second only to casualwear (Hollett & Challis, 2023). Estimates also suggest that 80% of women in Australian samples browse for apparel every month, with roughly an hour of their monthly browsing time spent specifically browsing for activewear (Hollett & Challis, 2023).

Meta-analytic evidence also points toward a high engagement with social media amongst women, with studies suggesting that at least half of US women visit social media platforms every day (Bae et al., 2015; Hruska & Maresova, 2020). Research also shows that women are well connected and responsive to fashion labels on social media (Bharti, 2021; Djafarova & Bowes, 2021; Nash, 2019; Seekis et al., 2020), with some estimates suggesting Australian activewear brands have at least 16 million followers across Facebook, Instagram, and TikTok (Hollett et al., 2024). Given the high rates of apparel browsing and social media engagement amongst women, we assume they are readily exposed to activewear imagery online.

### 1.2. Potential Positive Outcomes of Activewear Engagement

One potential explanation for the popularity of activewear is the success of marketing, which convinces women that activewear motivates them to be physically active and to feel more confident about their bodies (Lipson et al., 2020). As women’s overall self-esteem is closely linked to their body satisfaction and appearance attitudes (Adams et al., 2017; Moya-Garófano & Moya, 2019), fashion choices likely play a substantial role in maintaining women’s confidence in their appearance and generally feeling good about themselves (Kaiser, 1997). Importantly, qualitative evidence suggests that wearing activewear elicits feelings of athleticism and confidence among women (Brice et al., 2022; Lipson et al., 2020). Clothing that enhances the appearance and feel of one’s body, whilst exhibiting the aesthetic results gained through fitness behaviors (e.g., lean and toned muscle), may reinforce women’s self-worth and sense of confidence across various settings. Women may also wear activewear outside of exercise settings to showcase to their broader social network that they are making efforts to stay in shape. That is, wearing activewear could be a social signal that women eat well, exercise, and generally feel confident about their bodies (Barnard, 2013; Horton et al., 2016; Lipson et al., 2020).

Exposure to activewear imagery online, which advocates for an active lifestyle, healthy eating, and purposeful exercise, may also offer benefits to women. Depictions of healthy and toned women wearing activewear offer an exemplar that could motivate women to engage in deliberate efforts to enhance their well-being through healthy eating and increased exercise (Perera et al., 2024; Zhou, 2018). Indeed, healthy lifestyle orientation measures related to adopting a nutritional diet and regular exercise have been found to predict higher activewear purchasing intentions amongst US activewear consumers (Watts & Chi, 2019). Importantly, healthy eating and exercise are linked with various psychological benefits, including lower depression, anxiety, and stress (Bremner et al., 2020; Penedo & Dahn, 2005; Stanton et al., 2014; Warburton et al., 2006). Regular engagement in fitness activities may also operate as a protective factor against body image concerns (Alleva et al., 2019; Calogero et al., 2019), potentially buffering women from some of the appearance-related pressures common in activewear marketing. As such, activewear marketing could contribute meaningfully to the psychological well-being of women simply by promoting beneficial lifestyle choices.

If engaging with activewear does promote beneficial outcomes with respect to physical activity, body confidence, and self-worth, then we would expect positive associations between activewear engagement and fitness behaviors, body appreciation, and self-esteem. Indeed, many activewear brands utilize marketing messages that point toward these beneficial outcomes for women (Horton et al., 2016; Luna Mora & Berry, 2021; Ryderwear, n.d.-a; Zhou, 2018), yet very little peer-reviewed evidence exists to support these industry presumptions (Lipson et al., 2020). In the interests of informing female consumers and the activewear industry, we sought to determine if there are positive associations between activewear engagement and beneficial outcomes for women, such as increased fitness activity and body appreciation. As with any cultural trend reaping large financial rewards from consumers, the potential benefits must be weighed against the potential risks. Already, a substantial body of literature exists to inform activewear consumers and industry about the potential adverse outcomes for women who are heavily engaged with products and imagery that highlight the value of women’s bodies (Huang et al., 2021; Slater & Tiggemann, 2015; Vandenbosch & Eggermont, 2012; Vandenbosch et al., 2022). However, industry and consumers may need stronger persuasion through research specifically showing links between women’s activewear habits and potential negative outcomes for women.

### 1.3. Potential Negative Outcomes of Activewear Engagement

A notable modern trend of activewear includes tight, revealing styles promoted via sexualized postures in young athletic women (Hollett & Challis, 2023; Luna Mora & Berry, 2021). That is, youth and muscle tone are often promoted as key markers of female worth by activewear retailers (Brice et al., 2022; Luna Mora & Berry, 2021). Through the biased visual marketing of their products, it has been reported that some brands imply that only women who fit a particular body type are worthy of wearing their products (Brice et al., 2022; Lipson et al., 2020). Indeed, women have reported a tension between the athletic ideals portrayed by activewear and the perception of their own bodies, including the capacity of activewear to highlight their own physical flaws (Lipson et al., 2020). The overrepresentation of idealized body image standards in activewear marketing can be challenged by decades of research demonstrating that women’s psychological and physical well-being is threatened by the promotion of such standards (Huang et al., 2021; Vandenbosch et al., 2022). Importantly, exposure to sexually objectifying media can increase self-reported self-surveillance behaviors, negative mood, body shame, and anxiety and decrease body satisfaction (Hogue et al., 2023; Prichard et al., 2018; Robinson et al., 2017).

Increasingly sexualized styles of activewear also elevate the risk to women’s well-being via sexual objectification (Luna Mora & Berry, 2021; Vendemia et al., 2021). Sexual objectification involves representations of women that prioritize their body parts, particularly those with sexual relevance (e.g., buttocks, breasts, thighs), instead of their social, emotional, and intellectual characteristics (Fredrickson & Roberts, 1997; Hollett et al., 2022). As such, garments designed to highlight women’s bodies increase the risk that women will be objectified by others and that the wearer will objectify themselves (Felig et al., 2022; Greenleaf & Hauff, 2024). Self-objectification involves internalizing the view that one’s body and/or body parts are the primary attribute by which self-worth is determined (McKinley & Hyde, 1996; Moradi & Varnes, 2017). Habitual appearance monitoring and accompanying body shame are often considered markers of self-objectification (Moradi & Varnes, 2017).

Because women are particularly vulnerable to comparing themselves with other women (Myers & Crowther, 2009; Schaefer & Thompson, 2014), the widespread rise in activewear could be socially problematic, as it facilitates this tendency through several mechanisms. Firstly, form-fitting activewear styles allow and encourage women to more closely scrutinize their own and others’ body shape, more so than other forms of casualwear (Lipson et al., 2020; Luna Mora & Berry, 2021). Secondly, in a saturated market, brands utilize increasingly provocative strategies to secure consumer attention, which depend on unrealistic body image standards, sexualization, and an overemphasis on diet and exercise (Hollett et al., 2024). Thus, women are not only threatened by the physical comparison to other women but also by the lifestyle habits that apparently procure the kind of body best suited to activewear (Brice et al., 2022; Lipson et al., 2020). While social comparison theory explains that relative judgements against others are a natural phenomenon that offers some adaptive benefits (Buunk & Mussweiler, 2001; Festinger, 1954; Mussweiler & Epstude, 2009), unrealistic appearance and lifestyle standards promoted within the activewear industry create expectations and perceived discrepancies that undermine women’s well-being. Indeed, recent research has found that women with higher body shame and appearance comparisons have been shown to spend more money on activewear in a simulated shopping task (Hollett et al., 2023). Self-report evidence from larger online data collections also suggests modest links between increased time spent shopping for activewear and higher self-objectification (Hollett & Challis, 2023). Notably, decreases in women’s body image ratings following experimental exposure to activewear retail imagery are significantly associated with elevated disordered eating attitudes and higher engagement in body image coping strategies such as appearance fixing and avoidance (Hollett et al., 2024).

In addition to objectification and social comparison theories, self-perception theory offers a complementary perspective for understanding how clothing engagement may influence body image. Self-perception theory proposes that individuals infer attitudes about themselves by observing their own behaviors, similar to how an external audience might (Bem, 1972). Importantly, research on strategic self-presentation suggests that these behaviors may be especially influential when enacted publicly and repeatedly, as public commitment to a projected identity can produce enduring changes in self-appraisals and behavior (Schienker et al., 1994). From this perspective, wearing form-fitting or appearance-salient activewear across daily and social contexts creates a public identity signal and reinforces appearance-relevant standards to both observers and the self. Over time, this public commitment to an appearance-focused presentation may increase the salience of body evaluation, thereby shaping self-objectification tendencies and ideal internalization beyond immediate situational effects.

To date, there is growing evidence that increased time browsing for activewear, higher spending on activewear, and experimental exposure to activewear imagery are linked to adverse psychosocial and body image outcomes for women (Hollett et al., 2024; Hollett & Challis, 2023). However, less is known about the adverse impact of activewear wearing habits and social media engagement. It is also unclear whether activewear remains closely tied to exercise contexts and habits. Importantly, exercise clothing that promotes self-consciousness can discourage women from exercising (Greenleaf & Hauff, 2024), and women who wear loose clothing during exercise are less likely to be preoccupied with their appearance (Prichard & Tiggemann, 2005). Experimental evidence also shows that body monitoring habits may actually undermine the benefits of exercise if women pay attention to their bodies during fitness activities (Engeln et al., 2023). These findings challenge activewear brands that design garments to deliberately emphasize the appearance of women’s bodies, rather than prioritizing functional properties (Zhou, 2018).

When adhering to activewear trends, women risk putting on a display to others, which likely heightens their self-perceptions of body awareness. Needlessly heightened attention toward one’s body in any context can interfere with women’s capacity to perform and enjoy everyday tasks, including exercise (Engeln, 2017). Several studies support the assumption that women are vulnerable to adorning themselves in clothing that is distracting, uncomfortable, or restrictive across a variety of contexts (Engeln & Zola, 2021; Greenleaf & Hauff, 2024; Smolak et al., 2014; Tiggemann & Andrew, 2012). Evidence that women often prioritize style over function also points toward an established cultural issue whereby women are willing to objectify themselves to follow certain fashion trends, likely driven by media or social pressure (Felig et al., 2022; Lipson et al., 2020; Morganosky, 1984; Zhou, 2018).

In sum, preliminary evidence suggests that activewear is a fashion trend that may cause more harm than good (Brice et al., 2022; Lipson et al., 2020). Because activewear represents a cultural intersection between healthy habits (e.g., engaging in fitness behaviors) and unhealthy habits (e.g., adopting idealistic body standards), engaging with activewear raises a range of complex implications for women. However, the benefits and risks of activewear engagement amongst women are not well understood, and empirical research is needed to clarify how this rapidly growing industry might be affecting women’s well-being.

### 1.4. The Present Study

The present study was largely motivated by a lack of quantitative data available to properly understand the scale of activewear engagement amongst large samples of women. While qualitative research has pointed toward an *assumption* of wide engagement amongst women, and links to potential psychosocial constructs (Brice et al., 2022; Lipson et al., 2020), no study has offered a comprehensive descriptive account of women’s activewear engagement habits. Furthermore, given that activewear engagement cannot yet be confidently linked to primarily positive or negative outcomes for women, the present study was also designed to offer some clearer directions for understanding the impact of this fashion phenomenon. Firstly, we sought to describe the phenomenon by exploring women’s wearing, purchasing, browsing, and social media habits with respect to activewear. Secondly, we sought to estimate the correspondence between activewear habits and potentially positive outcomes, including fitness behaviors, body appreciation, and self-esteem. We also sought to estimate the correspondence between activewear habits and potentially negative outcomes, including media pressure, idealistic appearance aspirations, self-objectification, and self-consciousness while wearing activewear. As positive and negative outcomes are plausible in this context, we do not present directional hypotheses. Instead, we opted to explore associations amongst variables reflecting positive and negative psychosocial phenomena to guide future research on this issue. Conceptually, the variables examined in this study can be understood as operating across three interrelated domains: behavioral engagement with activewear, psychosocial appearance-related processes (media pressure, appearance comparisons), and body image attitudes (self-objectification, body/self-confidence, idealistic appearance aspirations). This organizing framework guided both the correlational and targeted mediation analyses reported.

While our statistical approach was largely exploratory, we recognized an opportunity to test more specific models to examine potential mediators that may strengthen links between activewear engagement and our chosen theoretical frameworks. Specifically, we explored the potential for media pressure and appearance comparisons to mediate potential associations between activewear engagement and body image indicators. Objectification theory suggests that engaging with body-accentuating clothing can heighten sensitivity to appearance-related media cues, thereby increasing the likelihood that women might internalize culturally promoted body ideals. Likewise, social comparison theory proposes that wearing appearance-revealing garments increases attention to others’ bodies, facilitating more frequent upward comparisons that also promote ideal internalization. Accordingly, in instances where media pressure and appearance comparison tendencies were significantly associated with both activewear engagement indicators and body image outcomes, we conducted targeted mediation analyses to examine whether these psychosocial processes might function as explanatory mechanisms.

## 2. Materials and Methods

### 2.1. Participants

Self-reported female participants (*N* = 829) were recruited from an undergraduate student population attending a broad-access Australian university (*N* = 455) and the Australian community (*N* = 374). The community samples were sourced via social media advertisements (*N* = 115) and a paid panel for a representative (age and location) Australian sample (*N* = 259). The student samples were aged between 18 and 65 years old (*M* = 28.38, *SD* = 10.18)^1^, and the community samples were aged between 18 and 90 years old (*M* = 44.10, *SD* = 16.05). In the student sample, most participants reported being Caucasian (77%), followed by Asian (8%), African (3%), or mixed/other (12%). In the community sample, most participants reported being Caucasian (82%), followed by Asian (10%), or mixed/other (8%). Of participants who provided weight data (89%), the average body mass index (BMI) was 25.32 (*SD* = 6.03) in the student sample and 28.17 (*SD* = 9.55) in the community sample. To help characterize the sample, a summary overview of participants’ engagement with activewear across both samples has been provided in Table 1.

### 2.2. Activewear Engagement Markers and Context Preferences

Participants were presented with questions to capture four distinct activewear engagement behaviors, used to provide a descriptive overview of women’s engagement with activewear as well as to explore associations with psychosocial and body image variables. Activewear was defined for participants as “clothing typically suitable for sport/gym, though also worn casually”. Firstly, participants were asked how many days per week on average in the last month they wore activewear on an eight-point response scale (from no days to seven days a week). They were also asked to estimate how much money ($AUD) they had spent on activewear over the last six months from 12 sequential monetary ranges (ranging from $0 to more than $5000). The midpoint of each monetary range was used to estimate a six-month spend score (e.g., $51–$100 was re-coded to $75). Participants were asked to estimate how many days per week (0–7) and minutes (15 min–8 or more hours) per day on average they spent browsing for women’s clothing online and to indicate the percentage of time that was spent on activewear. By multiplying the frequency (days) and the mid-point quantity (minutes) by the percentage data, a minutes-per-week estimate of activewear browsing time was calculated. Participants who reported wearing activewear in the last month were also asked to report how often they felt self-conscious when wearing activewear on a 5-point frequency scale anchored with “never”, “occasionally”, “roughly half the time”, “most of the time”, and “every time”. Finally, women were presented with an open text field and asked to list all the activewear brands they followed on social media. These were tallied to create an activewear brand following score for each participant.

Participants who reported wearing activewear in the last month were asked to estimate the percentage of time they wore activewear across six contexts using 0–100% sliders. The contexts were exercise (sport, gym, running, walking, etc.), running errands (groceries, appointments, etc.), attending social events, attending work, around the house/home, and attending school/university. Participants also had the option to allocate to and specify an “other” context. The percentages across all contexts were limited to sum to 100%.

### 2.3. Fitness Behaviors

Participants were asked to estimate how many hours per week they spent participating in fitness activities in the last month on average. Fitness activities were defined as “any activity which is a planned and purposeful behavior for improving fitness”. This measure was included to help understand the extent to which activewear engagement could be linked to beneficial physical activity.

### 2.4. Body Appreciation

The 10-item revised Body Appreciation Scale (BAS-2) (Tylka & Wood-Barcalow, 2015) measured the extent to which a person appreciates their own body and is considered a psychometrically sound measure of positive body image. Several studies have used this construct to explore links to fitspiration and body positivity imagery (Nelson et al., 2022; Slater et al., 2017; Tiggemann et al., 2020). This measure was included as a positively framed body image construct to determine if activewear engagement could be linked to body positivity, a common marketing theme promoted by activewear brands. Participants rated the items from 1 (never) to 5 (always). Composite scores were created by averaging the item ratings. Cronbach’s alpha for both samples was 0.96.

### 2.5. Self-Esteem

The 10-item Rosenberg Self-Esteem Scale (Rosenberg, 1965) measured trait self-esteem by capturing positive and negative feelings about the self. This self-esteem scale has been widely used to explore various body image effects (Adams et al., 2017; Tiggemann & Lacey, 2009). This measure was included as a general estimate of well-being in the present study to determine if activewear engagement could be linked to self-confidence and worth. Participants rated the items from 1 (strongly disagree) to 4 (strongly agree). Composite scores were created by summing the item ratings (range 10–40). Cronbach’s alpha across both samples was 0.89–0.90.

### 2.6. Idealistic Appearance Aspirations and Media Pressure

Three subscales (14 items) from the Sociocultural Attitudes Towards Appearance Questionnaire 4 (SATAQ-4; Schaefer et al., 2015) measured two components of idealistic appearance aspirations as well as perceptions of media pressure. Specifically, participants were asked to rate their desire to be thin with low body fat as well as their desire to be muscular/athletic. There were also four items related to the perceived impact of media pressure on appearance (e.g., I feel pressure from the media to look in better shape). This measure was included to understand the extent to which women feel pressure to adopt idealistic body norms and if these attitudes correspond with activewear engagement. Participants were asked to rate the items on a 5-point Likert scale anchored from 1 (definitely disagree) to 5 (definitely agree). Composite scores were created by averaging the item ratings for each subscale. The internal consistency estimates in both samples for thin/low-fat were 0.84–0.86; muscular/athletic were 0.91–0.92, and media pressure were 0.95–0.96.

### 2.7. Appearance Comparisons

The 11-item Physical Appearance Comparison Scale-Revised (PACS-R; Schaefer & Thompson, 2014) measured the tendency to compare one’s physical appearance to that of others. This measure was included to determine if women’s tendencies to engage in social comparisons correspond with activewear engagement. Participants rated the items from 0 (never) to 4 (always). Composite scores were created by averaging the item ratings. Cronbach’s alpha across both samples was 0.97–0.98.

### 2.8. Self-Objectification

Two subscales (16 items) from the Objectified Body Consciousness Scale (OBSC; McKinley & Hyde, 1996) measured the extent to which participants engage in surveillance of their own body and the shame they experience in relation to their body. This measure was included to determine if women who place undue value on their bodies are more likely to engage with activewear. Participants rated the items from 1 (strongly disagree) to 7 (strongly agree). Composite scores were created by averaging the item ratings for each subscale. Cronbach’s alpha across both samples for surveillance was 0.85–0.87 and 0.86–0.87 for body shame. The 7-item Provocative Gaze Behavior scale (Hollett et al., 2022) was also used to estimate the extent to which participants engage in behaviors (e.g., posture, revealing clothing) that draw attention to their body. This measure offered another means to understand the association between self-objectification habits and activewear engagement in women. Cronbach’s alpha across both samples was 0.89–0.95.

### 2.9. Data Collection Procedure and Analysis

Links to the online survey were distributed via three different recruitment channels (a university participation scheme, social media, and via a paid panel). Following the provision of consent, participants completed demographic and online shopping questions. The psychosocial and body image measures (body appreciation, self-esteem, self-objectification, idealized appearance aspirations, media pressure, and appearance comparisons) were then presented in a randomized order, followed by the remaining activewear engagement items, and a fitness hours estimate. Note that only women who reported wearing activewear in the last month were presented with the activewear context questions and the self-conscious-while-wearing-activewear question. The survey took 15–20 min and, depending on the recruitment channel, participants were offered course credit, a panel incentive, or entry into a gift card draw on completion. These procedures were approved by the university’s human research ethics committee.

Descriptive statistics and distributions were calculated and reported separately by sample for each of the activewear engagement markers. Zero-order intercorrelations were then reported between all the quantitative variables using *p* = 0.01 as a threshold for significance to mitigate potential type I error, with *p* values estimated via bootstrapping so that no distribution assumptions were required. The Statistical Package for Social Sciences (v29) was used to perform initial analyses (IBM, 2024), and Jamovi (v2.6) was used to perform (bias-corrected) bootstrapped mediation analyses (Jamovi, 2025).

## 3. Results

### 3.1. Data Screening

Following the exclusion of incomplete responses, the data were screened to remove cases that failed attention checks (*N* = 53) or spent too little (less than 5 min) or too long (more than 60 min) on the survey (*N* = 51). This left 829 cases for analysis across both samples. Most data were normally distributed except for six-month spend and browsing minutes per week. However, medians are reported for all variables, and bootstrapping was used to estimate significance for all statistical tests (which does not require distribution assumptions).

### 3.2. Activewear Engagement Descriptives

**Figure 1:**
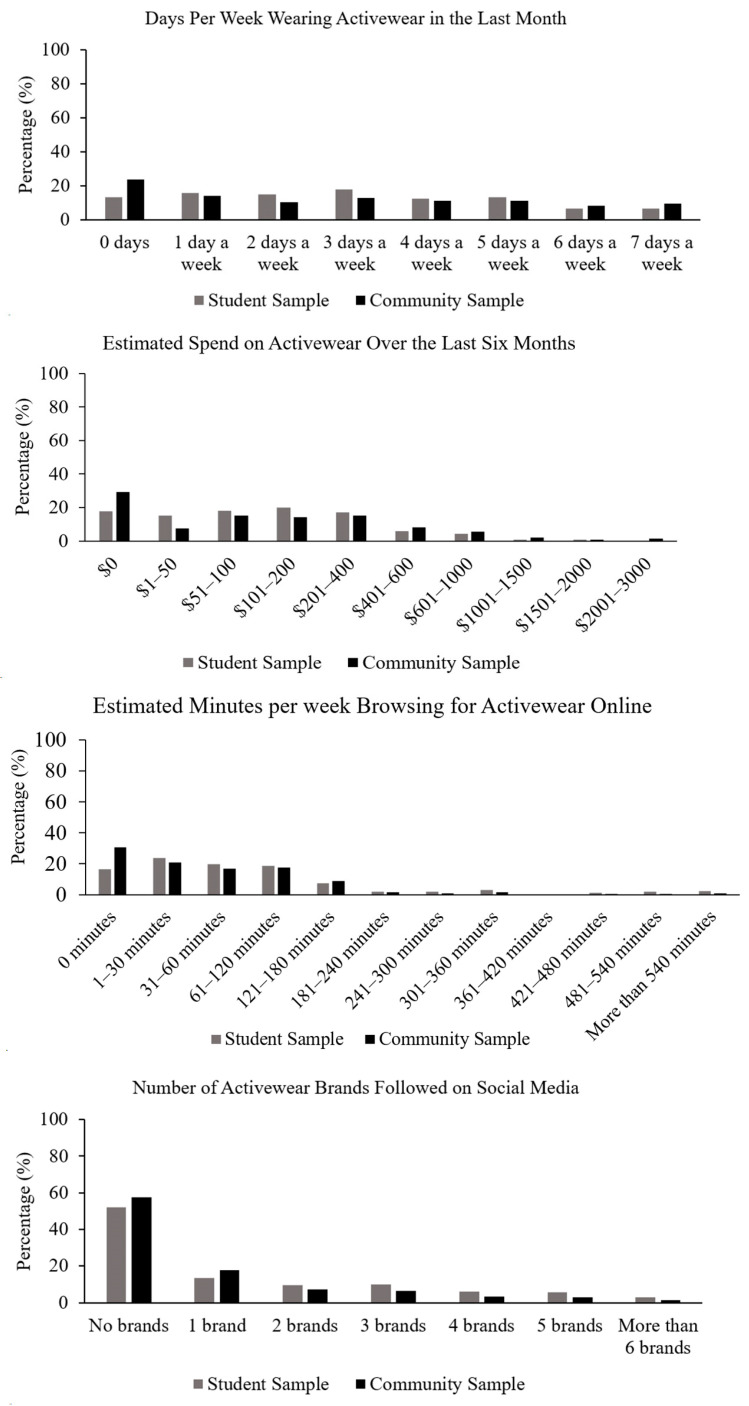
Distributions for the markers of activewear engagement.

The descriptive statistics for the markers of activewear engagement have been reported in Table 2. Both samples reported a similar number of wearing days per week (*p* = 0.443) and online browsing minutes per week (*p* = 0.920), but there were significant differences between six-month spend (*p* = 0.019) and brand following (*p* = 0.002). To complement the descriptive statistics, distributions for each engagement marker have been reported in Figure 1.

**Figure 2:**
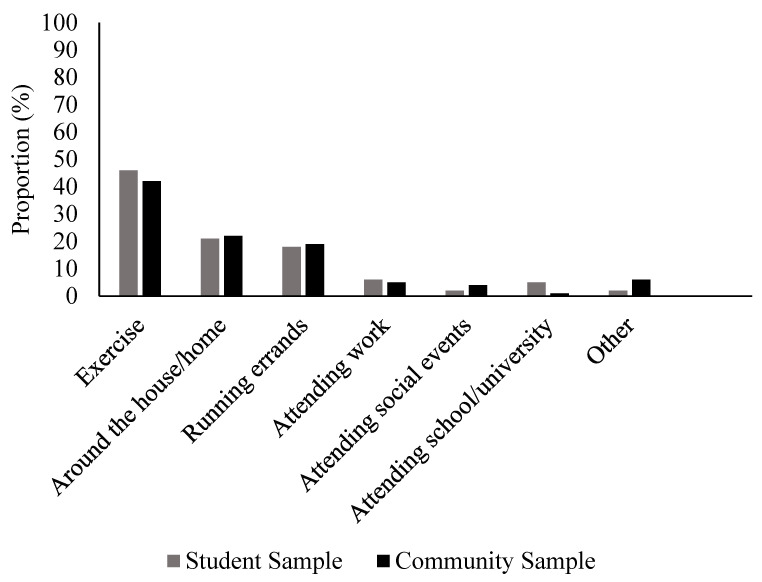
Estimated average proportion of total wearing time that activewear is worn in different contexts.

To understand the contexts in which activewear is worn, Figure 2 reports the estimated average percentage of time that activewear is worn in different contexts amongst women who reported wearing activewear in the last month (*N* = 680). Importantly, women reported that activewear was worn for exercise less than 50% of the time on average across both student (*M* = 46.50%, *SD* = 31.44) and community (*M* = 41.21%, *SD* = 33.21) samples. Furthermore, across both samples, only 10.14% of women reported only wearing activewear for exercise (with no other contexts).

### 3.3. Associations Between Activewear Engagement Markers and Positive Outcomes

As can be seen in Table 3, activewear wearing and six-month spend correlated significantly and positively with fitness hours in the student sample, while all the activewear engagement markers correlated significantly and positively with fitness hours in the community sample. However, none of the activewear engagement markers correlated with body appreciation or self-esteem in either sample.

### 3.4. Associations Between Activewear Engagement Markers and Negative Outcomes

**Figure 3:**
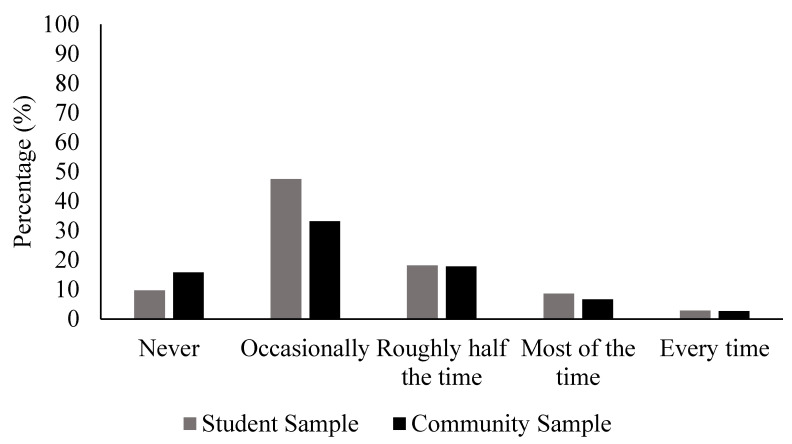
Distribution of responses regarding how often women who wear activewear feel self-conscious about their body.

As can be seen in Table 3, activewear wearing, six-month spend, and brand following correlated significantly and positively with muscular/athletic aspirations in the student sample. In the community sample, all the activewear engagement markers correlated significantly and positively with muscular/athletic aspirations. Also, in the community sample, activewear wearing and six-month spend were significantly and positively correlated with thin/low-fat aspirations and appearance comparisons. Activewear wearing was also correlated significantly and positively with media pressure and surveillance in the community sample. There were no significant associations between the activewear engagement markers and shame or body gaze provocation in either sample. The distribution of the activewear self-conscious responses amongst women who reported wearing activewear at least one day per week in the last month (N = 680) has been provided in Figure 3. Importantly, almost 30% of these women reported feeling self-conscious half the time or more.

### 3.5. Mediation Analyses

Consistent with our theoretical rationale for exploring targeted mediation models, we identified four suitable models that met the traditional criteria for conducting a mediation analysis (Baron & Kenny, 1986). As the proposed mediators of media pressure and appearance comparisons were only significantly correlated with activewear engagement (wearing only) in the community sample, we tested the following models in this sample only; (1) media pressure as a mediator between activewear wearing and thin/low-fat aspirations; (2) media pressure as a mediator between activewear wearing and muscular/athletic aspirations; (3) appearance comparisons as a mediator between activewear wearing and thin/low-fat aspirations; (4) appearance comparisons as a mediator between activewear wearing and muscular/athletic aspirations. The results of these analyses using standardized effects have been reported in Table 4.

As can be seen in Table 4, both media pressure and appearance comparisons yielded significant indirect effects on the association between activewear wearing and both thin/low-fat and muscular/athletic body image ideals. However, across both sets of media pressure and appearance comparison mediation models, the strongest mediation effects emerged when the outcome variable was thin/low-fat body image ideals.

## 4. Discussion

The purpose of this study was to provide a comprehensive exploration of the activewear phenomenon amongst women. Specifically, we aimed to describe activewear engagement habits and examine links between activewear engagement, psychosocial, and body image risk factors across two samples of women. The main findings can be summarized in terms of the potential positive and negative links to activewear engagement. Firstly, a positive link to activewear engagement was supported by its association with more frequent fitness behaviors, suggesting that activewear encourages or reflects a motivation to stay physically active. Secondly, despite the link to fitness behaviors, we observed no links between activewear engagement and body appreciation or self-esteem. Thirdly, the negative links to activewear engagement included increased risk of self-objectification and body dissatisfaction, supported by associations with measures of heightened body awareness and unrealistic ideals. We offer a more detailed exploration of the results, which support these points below.

The descriptive data suggest high engagement with activewear amongst women in the two samples, with high proportions recently wearing (76–87%) and purchasing (67–82%) activewear. Digital engagement with activewear amongst the two samples was also notable, with 56–67% of women browsing for activewear online in the last month and 40–48% following at least one activewear brand on social media. We also provide quantitative evidence supporting prior assumptions that activewear is not predominantly worn in exercise settings, with wearing across other settings (errands, at home, social events, school/university) accounting for more than 50% of the time spent wearing activewear. Notably, we also find that only around 10% of the women across our samples *only* wore activewear in exercise settings, further illustrating that activewear has successfully transitioned into multiple contexts for a large proportion of women.

Importantly, we found no associations between activewear engagement and positive psychological outcomes (body appreciation and self-esteem). This contradicts activewear industry marketing as well as qualitative reports that activewear enhances body confidence and esteem (Brice et al., 2022; Gymshark, n.d.-a; Lipson et al., 2020). While links between activewear and both body appreciation and self-esteem were not evident in the current study, we did find a robust link between activewear engagement and fitness behaviors. The relatively large positive associations between weekly fitness hours and activewear engagement across both samples suggest that women who are engaged with activewear may also be experiencing the numerous mental and physical health benefits associated with physical activity (Bremner et al., 2020; Warburton et al., 2006). However, we found little evidence of fitness activity as a protective factor for negative body image in our data, as it did not positively correlate with self-esteem or body appreciation in either sample. It also did not negatively correlate with self-objectification, appearance comparisons, or idealized aspirations. Note that our fitness data were very limited, as they did not differentiate between types of exercise or exercise motivations, such as health-oriented or appearance-oriented. Indeed, some evidence indicates that mindful forms of exercise, such as yoga, may be more likely to serve a protective function (Calogero et al., 2019).

There were significant, large positive correlations between engaging with activewear and aspirations to be muscular and athletic across both samples and significant positive associations with aspirations to be thin with low body fat in the community sample only. The pattern of associations suggests that activewear may play a role in reinforcing unrealistic body standards among women, possibly through mechanisms involving pressure from the media. This assumption is supported by the strong correlations between perceived media pressure and both thin/low-fat and muscular/athletic aspirations in the community sample. Importantly, a relatively large positive association was observed between wearing activewear in the community sample and perceived media pressure. That is, women in the community sample who wore activewear more often were more likely to report that they felt pressure from the media to possess a better shape, look thinner, and improve their appearance. Indeed, the targeted mediation analyses provide further support for the role of media pressure by showing that both media pressure and appearance comparisons significantly indirectly explain the variance shared between activewear wearing and the internalization of body image ideals. These associations correspond with qualitative studies suggesting that activewear media sources promote petite body types with flat stomachs (Lipson et al., 2020) and that activewear offers an “invisible corset” (via controlling, compressing, and contouring fabrics and designs) which permits women the capacity to comply with these media-derived social norms (Luna Mora & Berry, 2021). Given the non-directional nature of our data, the statistical findings, together with qualitative reports, suggest a reciprocal pattern in which media pressure, appearance comparisons, and activewear engagement may reinforce each other.

Notably, and consistent with prior literature, we found significant positive associations between activewear engagement and appearance comparisons and body surveillance tendencies in the community sample (Hollett & Challis, 2023). The significant associations between idealized aspirations, media pressure, appearance comparisons, and surveillance in the community sample all point toward psychosocial motivations for engaging with activewear in women. Because these correlations clustered in a manner consistent with theoretical expectations, they suggest a coherent pattern rather than random effects. Furthermore, the finding that around 30% of women who wear activewear in our samples reported feeling self-conscious half the time or more aligns with our assumptions that wearing activewear elicits heightened body awareness. This distribution of self-consciousness responses reinforces the assumption that body awareness effects are not limited to a small subgroup but are likely evident at the population level. Importantly, our results highlight and confirm several key findings from recent qualitative studies. Firstly, activewear represents an embodiment of the fit ideal, with qualitative reports showing that women perceive activewear as a signal that the wearer values fitspiration (Lipson et al., 2020), supported further by our quantitative data showing strong associations between wearing activewear and muscular/athletic aspirations. Secondly, participants in several qualitative studies (Brice et al., 2022; Lipson et al., 2020) identify activewear as facilitating a “postfeminist gaze” which imposes positive and negative body image standards on oneself and others. These sentiments are argued to create a paradox whereby activewear could reflect an expression of empowerment but also lead to feeling exposed and judged (Luna Mora & Berry, 2021). Similarly, our data show that women who wear activewear may be empowered via engagement in fitness activities and adhering to their athletic aspirations, but simultaneously experience increased self-surveillance, appearance comparisons, and self-consciousness. Taken together, our study continues to support the emerging literature that activewear engagement is linked to adverse outcomes for women, likely driven by self-objectification and social comparison processes (Brice et al., 2022; Hollett et al., 2024; Luna Mora & Berry, 2021).

### 4.1. Theoretical and Practical Implications

We found more evidence that activewear engagement is linked to negative factors rather than positive factors. If activewear was an effective fashion item for eliciting body confidence and flattery, then we would have observed positive associations between activewear engagement and both body appreciation and self-esteem. Furthermore, the large association between wearing activewear and media pressure, as well as the large proportion of women wearing activewear outside of exercise settings, suggests that activewear brands have successfully persuaded many women that activewear is a comprehensive fashion solution across a variety of contexts, despite the risks to body image. In our community sample, we observed that younger women are more likely to engage with activewear, which likely reflects the effectiveness of marketing imagery used by activewear brands for targeting young female consumers. One implication of this is that younger women might feel greater pressure to engage with activewear and experience adverse body image outcomes. To explore this further, using the median age to split the community sample, we examined our correlations separately for women aged under 40 years of age and those aged 40 years or more (provided in Table S1 of the Supplementary Materials). Contrary to our assumption, the associations between activewear engagement and media pressure were larger and significant in the older age group. In fact, many of the associations between activewear engagement and body image variables were only significant in the older age group, suggesting that older women who engage with activewear are more likely to report elevated appearance comparison and self-objectification attitudes. This raises the possibility that body image among older women might be under greater threat when they engage with activewear relative to younger women.

Similarly, we also explored the potential moderating effect of body mass index as classified by the Department of Health, Disability and Ageing (2026). Because activewear marketing imagery tends to prioritize models reflecting lower BMI classifications, a measure related to body size may be valuable for determining women’s vulnerability to the psychosocial and body image impacts of activewear engagement. As can be seen in Table S2 of the Supplementary Materials, women classified as healthy or underweight (BMI < 25) showed stronger and more consistent associations between activewear spending and both appearance comparisons and self-objectification, compared to women classified as overweight or obese (BMI > 24). By contrast, activewear wearing was more consistently associated with appearance comparisons and self-objectification in the overweight group, though modestly. Notably, associations between muscular/athletic aspirations were consistently linked to activewear engagement in both BMI groups, but thin/low-fat aspirations were only linked to activewear engagement in the lower BMI group. These results suggest that age and BMI are important individual differences for understanding how women’s engagement with activewear can vary, which is helpful for informing risk profiles across different forms of engagement. For instance, older women may be at greater risk of reporting adverse psychosocial and body image attitudes when wearing and purchasing activewear. By contrast, activewear spending behavior in younger women and wearing behaviors in older women may reflect differentially useful markers of body image risk when engaging with activewear.

From a theoretical standpoint, our results further illustrate the relevance of both objectification and social comparison theories for explaining the risks associated with clothing styles that enhance the sexualization of women, unnecessarily increase awareness of their own bodies and facilitate appearance comparisons (Festinger, 1954; Moradi & Varnes, 2017). Indeed, both theories highlight relevant psychosocial mechanisms such as internalizing a worldview that one’s body contributes substantially to self-worth and how deviations from a culturally derived body type norm (e.g., low fat, athletic) can elicit perceived discrepancies when engaging in upward comparisons to peers (Moradi & Huang, 2008; Myers & Crowther, 2009). Specifically, objectification theory positions self-surveillance, body monitoring, and internalized body evaluation as predictable responses when clothing emphasizes the body, which aligns with our findings linking activewear engagement to heightened body awareness and self-consciousness. By contrast, social comparison theory predicts that frequent exposure to idealized bodies creates increased opportunities for upward comparisons, which is reflected in the associations we observed between activewear engagement, appearance comparison tendencies, and idealized body aspirations. Importantly, our targeted mediation analyses add further weight to the assumed theoretical mechanisms that underlie the internalization of idealized body image. That is, media pressure and appearance comparisons explained similar amounts of variance between activewear engagement and idealized body standards, reinforcing the explanatory value of objectification and social comparison theories. The strongest mediation effects emerged for thin and low-fat ideals, indicating that these processes may be especially potent drivers of thinness-oriented aspirations. Importantly, these mediation models illustrate that activewear engagement has implications for self-perceptions, particularly when activewear behaviors are publicly visible (e.g., wearing activewear) because this may signal a stronger commitment to a public identity, which reinforces body evaluation tendencies. Ultimately, the activewear industry is a compelling example of how women’s vulnerability to engage in upward comparisons and adopt self-objectifying attitudes can be exploited for securing both attention and revenue.

Our results also support a growing body of research suggesting that activewear retailers take greater responsibility for the impact of sexually objectifying marketing strategies on women’s psychological well-being (Hollett et al., 2024; Lipson et al., 2020). Specifically, our results indicated positive associations between activewear engagement, appearance comparisons, and body surveillance and that many women often felt self-conscious wearing activewear, likely due to tight and revealing designs. We argue that trends in fashion may play a substantial role in perpetuating sexual objectification at the societal level despite poor empirical links to increased purchasing behavior (Bae et al., 2015; Gramazio et al., 2021). For example, Bae et al. (2015) examined the portrayal of women as sexual objects in the marketing for two prominent fashion brands and found that sexually objectifying advertising did not positively impact purchase intentions. Similarly, Gramazio et al. (2021) found that women report higher purchase intentions when advertisements do not contain sexualized imagery compared to advertisements with sexualized imagery. Whilst some brands may continue to use sexually objectifying imagery to promote activewear, the evidence cited above suggests that brands can adopt less sexualized imagery and remain financially lucrative.

One practical implication emerging from our research is the responsibility of leading brands to align their marketing and product strategies with changing social values, particularly in the areas of body positivity and diverse representation. These are important objectives given the considerable financial benefits currently observed within the activewear industry. For instance, one Australian brand, LSKD, recently announced a $100 million turnover in a single year, and anticipated turnover targets of $400 million by 2039 and $1 billion by 2032 (LaRenz, 2024). Given that there are almost 100 activewear brands in Australia alone (Hollett et al., 2024), the industry is making significant profits, particularly from women (Statistica, 2025, 2026). Given the increasing prevalence of activewear and emerging research suggesting negative effects of activewear imagery on women’s self-esteem and body image (Hollett et al., 2024; Hollett & Challis, 2023), it is important to consider existing studies that emphasize the benefits of fostering a positive body image for improved psychosocial well-being (Alleva et al., 2023; Cohen et al., 2019). This disconnect between prominent activewear brands’ advertising and research suggests that, despite a societal shift toward body acceptance, evident in the expanding #bodypositivity movement on social media, current activewear marketing and design may not reflect these values (Cohen et al., 2019). Women now actively challenge idealized standards by sharing images in unflattering angles or lighting, often in activewear, to promote body acceptance and appreciation (Walker, 2024). This movement away from externally imposed ideals shows that some consumers favor advertising that embodies diversity, confidence, and freedom from societal judgment (Alleva et al., 2023). For brands, this shift underscores the need to recalibrate imagery and messaging to genuinely support inclusivity and respect, rather than superficially adhering to social norms inconsistently. Adopting these positive values authentically is one possible strategy for brands to build lasting consumer trust and loyalty (Portal et al., 2019). However, those that ignore or superficially engage with these issues risk potential losses in market position as consumers prioritize acceptance and diversity to protect their well-being. Continued research aimed at understanding the impact of this highly lucrative fashion phenomenon on the well-being of women is one mechanism to maintain the accountability of activewear brands to their consumers.

### 4.2. Limitations and Future Directions

While the current study offers a valuable contribution toward understanding a widespread fashion phenomenon that intersects with women’s well-being, we recognize several limitations. Firstly, our data were collected online under uncontrolled conditions, which may have introduced measurement error. However, online data collection was necessary to reach a wide cross-section of respondents, which was an important objective to improve upon prior studies relying on laboratory data collections and/or only student samples (e.g., Hollett et al., 2024). We mitigated this limitation by utilizing multiple attention checks and completion time thresholds to reduce the potential influence of problematic data. Secondly, while we attempted to gather a large sample of respondents from different recruitment sources, our study was vulnerable to self-selection biases, whereby women who hold homogenous views toward activewear may be more likely to respond. For instance, one unexpected finding was that there was no association between activewear engagement and body shame, which has been established in prior laboratory studies (Hollett et al., 2024). One possibility is that women who experience higher levels of body shame may have been less inclined to complete a survey on activewear habits because of the implied link between fitness and appearance. Thirdly, while our results offer useful descriptive information and correlational guidance toward potential predictors of activewear engagement, our study was limited in explaining why activewear engagement has become somewhat ubiquitous amongst women. Finally, we recognize that results obtained through a correlational design prevent conclusions about whether activewear engagement positively or negatively affects women. However, when interpreted alongside several recent experimental studies showing similar links between activewear engagement and adverse body image factors (Hollett et al., 2024; Hollett & Challis, 2023), our data do support the assumption that these effects may extend beyond smaller samples tested under controlled conditions.

One potential avenue for future research would be to secure respondents from specific population subgroups, such as gym-goers or athletes, to better understand the role activewear plays among those who regularly engage in fitness behaviors and belong to communities that are highly engaged with activewear. It might also be useful to distinguish between users of different exercise contexts, such as fitness studios versus team or individual sports, as there may be important sociocultural variations in how fitness apparel is perceived across these contexts. For instance, some fitness venues contain large mirrors, which may facilitate appearance comparisons and self-objectification while wearing activewear. While our descriptive and habitual data provide an important foundation for understanding activewear engagement, we encourage future researchers to examine the motivations driving these behaviors to further clarify activewear engagement as a psychological outcome.

## 5. Conclusions

The present study is an important step toward understanding activewear engagement and the potential benefits and risks at the individual differences level in a large sample of Australian women. Australia is a valuable exemplar of a nation containing an activewear industry with substantial recent growth and a high level of adoption amongst women. As such, our data may translate well to contexts in which similar fashion trends have emerged. We anticipate that the evidence presented here will be valuable to a range of scholars, consumers, and activewear brands in global first-world contexts. Importantly, our data further support the assumption that the activewear phenomenon is a sociocultural lynchpin between fashion, body image standards, exercise, and the objectification of women. While correlational research is limited in drawing conclusions regarding the causal effects of fashion on consumer well-being, there is now mounting evidence that exposure to activewear puts women at risk of diminished body image via several psychological processes.

## Figures and Tables

**Table 1:** Overview of sample engagement with activewear.

Activewear Engagement	Sample	
	Student	Community
Worn in the last month	87%	76%
Purchased in the last 6 months	82%	71%
Browsed in the last month	67%	56%
Follow brands on social media	48%	40%

**Table 2:** Descriptive statistics for the markers of activewear engagement.

	Student Sample		Community Sample	
Activewear Engagement	Mean (SD)	Median	Mean (SD)	Median
Wearing Days	2.98 (2.06)	3.00	2.86 (2.36)	3
Six Months Spend ($) *	189.84 (272.72)	75	251.54 (442.77)	75
Online Browsing	21.11 (94.67)	4.50	20.60 (48.80)	3.26
Brand Following *	1.38 (1.91)	0	0.95 (1.62)	0

Note. Wearing days was measured in days per week; six-month spend was measured in AUD; online browsing was measured in minutes per week; brand following was measured by counting named brands followed on social media. * Significant mean differences between samples (*p* < 0.05).

**Table 3:** Zero-order intercorrelations between activewear engagement markers and potential positive and negative psychosocial and body image factors.

**Student Sample (*N* = 455)**															
	**1.**	**2.**	**3.**	**4.**	**5.**	**6.**	**7.**	**8.**	**9.**	**10.**	**11.**	**12.**	**13.**	**14.**	**15.**
1. Activewear Wearing	-														
2. Six-Month Spend	**0.44**	-													
3. Online Browsing	0.15	0.13	-												
4. Brand Following	**0.30**	**0.31**	0.11	-											
5. Fitness Hours per Week	**0.36**	**0.31**	0.05	0.18	-										
6. Media Pressure	0.04	0.05	0.09	0.10	−0.07	-									
7. Thin/Low-Fat Aspirations	0.06	0.06	0.08	0.09	0.01	**0.46**	-								
8. Muscular/Athletic Aspirations	**0.49**	**0.21**	0.10	**0.28**	**0.27**	0.10	**0.32**	-							
9. Self-Esteem	0.05	0.11	−0.04	0.07	0.06	**−0.36**	**−0.32**	−0.05	-						
10. Body Appreciation	0.03	0.07	−0.05	0.06	0.11	**−0.41**	**−0.39**	0.06	**0.74**	-					
11. Appearance Comparisons	0.06	0.07	0.05	0.11	−0.01	**0.57**	**0.40**	**0.21**	**−0.45**	**−0.52**	-				
12. Surveillance	0.05	0.04	0.03	0.15	−0.02	**0.47**	**0.50**	0.14	**−0.44**	**−0.53**	**0.64**	-			
13. Shame	0.08	0.04	0.08	0.09	−0.03	**0.48**	**0.51**	**0.18**	**−0.63**	**−0.62**	**0.63**	**0.56**	**-**		
14. Body Gaze Provocation ^#^	0.00	0.08	−0.04	0.01	0.02	0.12	0.15	**0.19**	−0.14	−0.03	**0.18**	0.17	0.14		
15. BMI ^†^	−0.05	0.05	−0.03	−0.01	−0.07	**0.20**	−0.06	**−0.18**	−0.10	**−0.27**	**0.21**	0.00	**0.21**	−0.06	
16. Age	−0.01	0.08	0.00	−0.06	0.04	−0.11	**−0.28**	−0.12	**0.27**	0.13	−0.16	**−0.21**	−0.17	−0.14	**0.23**
**Community Sample (*N* = 374)**															
1. Activewear Wearing	-														
2. Six-Month Spend	**0.42**	-													
3. Online Browsing	**0.30**	**0.38**	-												
4. Brand Following	**0.44**	**0.48**	**0.27**	-											
5. Fitness Hours per Week	**0.43**	**0.32**	**0.21**	**0.36**	-										
6. Media Pressure	**0.32**	0.19	0.10	0.17	0.15	-									
7. Thin/Low-Fat Aspirations	**0.25**	**0.20**	0.15	0.13	0.16	**0.56**	-								
8. Muscular/Athletic Aspirations	**0.45**	**0.38**	**0.27**	**0.35**	**0.26**	**0.41**	**0.50**	-							
9. Self-Esteem	−0.01	−0.11	0.08	−0.01	0.03	**−0.38**	**−0.40**	−0.10	-						
10. Body Appreciation	0.03	0.04	−0.02	0.02	0.10	**−0.31**	**−0.26**	0.13	**0.74**	-					
11. Appearance Comparisons	**0.24**	**0.20**	0.18	0.16	0.11	**0.60**	**0.55**	**0.36**	**−0.48**	**−0.38**	-				
12. Surveillance	**0.29**	0.14	0.13	**0.22**	0.12	**0.56**	**0.50**	**0.22**	**−0.44**	**−0.45**	**0.63**	-			
13. Shame	0.15	0.12	0.11	0.05	0.06	**0.53**	**0.52**	**0.29**	**−0.63**	**−0.56**	**0.58**	**0.54**	**-**		
14. Body Gaze Provocation *	0.08	0.18	0.09	−0.06	0.01	0.16	**0.27**	**0.45**	−0.13	0.09	**0.30**	0.05	**0.25**		
15. BMI ^‡^	−0.16	−0.03	−0.04	−0.08	−0.07	0.02	0.02	−0.12	−0.16	**−0.25**	0.03	−0.06	**−0.19**	0.11	
16. Age	**−0.41**	**−0.28**	−0.16	**−0.32**	**−0.21**	**−0.44**	**−0.39**	**−0.48**	0.17	0.01	**−0.35**	**−0.35**	**−0.23**	**−0.19**	0.11

Bootstrapping was used to estimate all *p*-values. Significant correlations (*p* < 0.01) are reported in bold. BMI = body mass index. ^#^ *N* = 425, ^†^ *N* = 411, * *N* = 360, ^‡^ *N* = 329.

**Table 4:** Mediation models for activewear wearing on body image ideals via media pressure (MP) and appearance comparisons (AC) in the community sample.

Model	Indirect Effect (X → M → Y)	Direct Effect (X → Y)	Total Effect	% Mediated by Indirect Effect
1 (MP)	β = 0.17 **	β = 0.08	β = 0.25 **	68%
2 (MP)	β = 0.10 **	β = 0.35 **	β = 0.45 **	22%
3 (AC)	β = 0.12 **	β = 0.13 *	β = 0.25 **	48%
4 (AC)	β = 0.07 **	β = 0.38 **	β = 0.45 *	16%

* *p* < 0.01, ** *p* < 0.001.

## Data Availability

The data have been provided as part of the Supplementary Materials.

## Note

1 Note that the student sample contains a high proportion of mature-age students (age 25 years and over). Histograms and percentage tables for age data in both samples have been provided in the Supplementary Materials.

## Supplementary Materials

The following supporting information can be downloaded at: https://www.mdpi.com/article/10.3390/bs16040586/s1, Figure S1: Distributions and Proportions of Age Values Across Both Samples; Table S1: Zero-Order Intercorrelations between Activewear Engagement Markers and Potential Positive and Negative Psychosocial and Body Image Factors, Separated by Age Group in the Community Sample; Table S2: Zero-Order Intercorrelations between Activewear Engagement Markers and Potential Positive and Negative Psychosocial and Body Image Factors, Separated by Body Mass Index Groups in the Community Sample.
