# Supplementary material for: The Age of Activewear: Understanding Women’s Casualized Athletic Apparel Habits Through Associations with Psychosocial and Body Image Factors
Source: Behav Sci (Basel). 2026 Apr 14;16(4):586. doi: 10.3390/bs16040586 (PMC13114242; doi:10.3390/bs16040586)
Supplement: Supplementary file 1 [file behavsci-16-00586-s001.zip › Supplementary Materials_R.pdf]

Figure S1. Distributions and Proportions of Age Values Across Both Samples

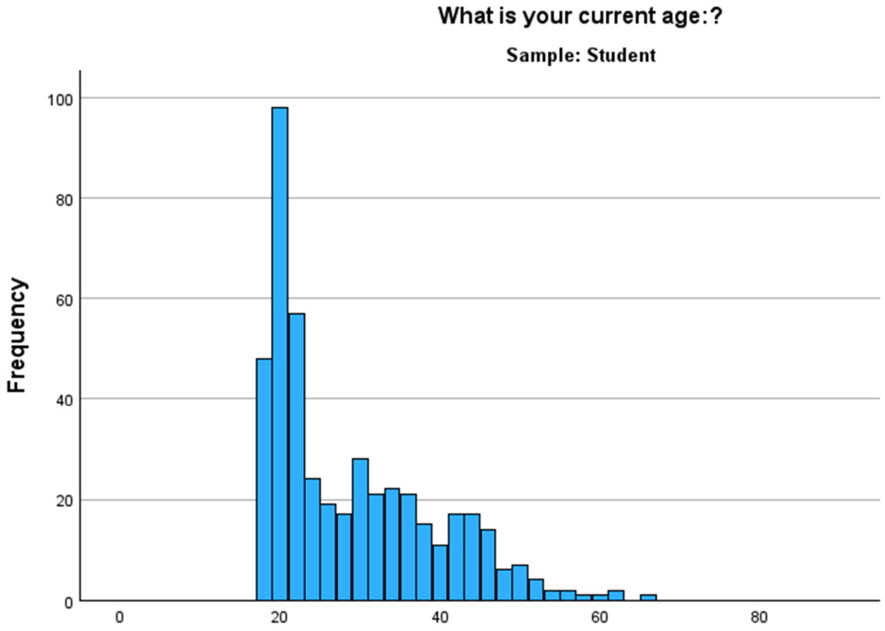

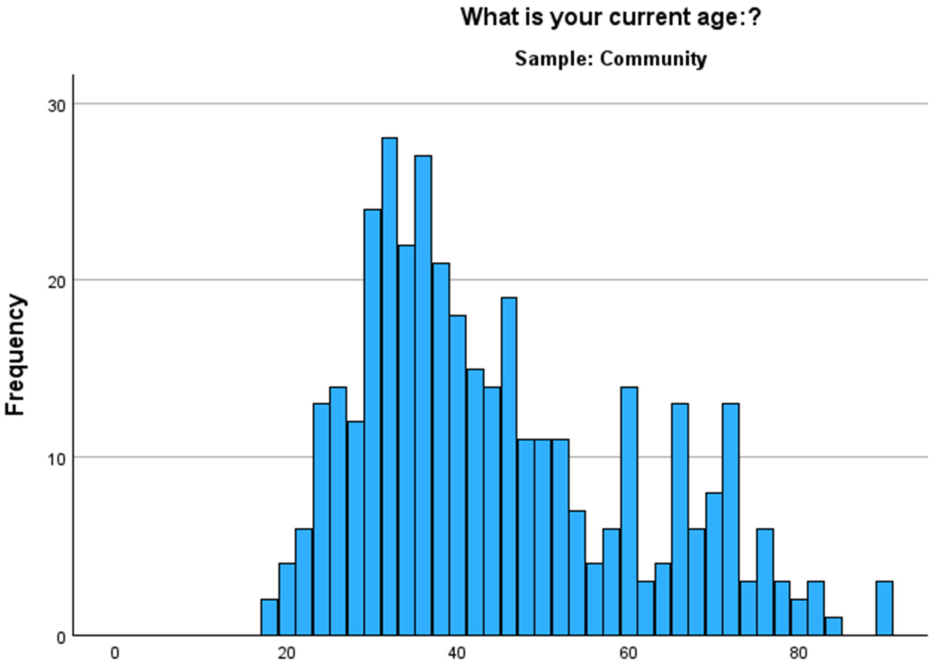

Age range	Student Sample	Community Sample
18-25 years	52%	10%
26-35 years	24%	27%
36-45 years	18%	25%
46-55 years	5%	14%
Above 55 years	1%	24%

Table S1. *Zero-Order* Intercorrelations between Activewear Engagement Markers and Potential Positive and Negative Psychosocial and Body Image Factors, *Separated by Age Group in the Community Sample.*

	1.	2.	3.	4.	5.	6.	7.	8.	9.	10.	11.	12.	13.
**Women aged < 40 years**													
1. Activewear Wearing	-												
2. Six Months Spend	**.33**	-											
3. Online Browsing	**.30**	**.54**	-										
4. Brand Following	**.37**	**.48**	**.27**	-									
5. Fitness Hours p/week	**.33**	**.21**	.17	**.27**	-								
6. Media Pressure	.04	.03	.11	.05	.10	-							
7. Thin/Low Fat Aspirations	-.02	.12	.16	.02	.07	**.37**	-						
8. Muscular/Athletic Aspirations	**.35**	**.29**	**.28**	**.30**	.17	.19	**.32**	-					
9. Self-Esteem	.17	-.11	-.04	-.06	.06	**-.26**	**-.37**	-.04	-				
10. Body Appreciation	.07	.00	.02	-.09	.07	**-.29**	**-.42**	.15	**.74**	-			
11. Appearance Comparisons	-.03	.06	.14	.06	.06	**.53**	**.47**	**.23**	**-.44**	**-.39**	-		
12. Surveillance	.06	.01	.06	.17	.03	**.51**	**.47**	.00	**-.36**	**-.49**	**.55**	-	
13. Shame	-.01	.02	.06	.01	-.01	**.43**	**.49**	**.17**	**-.57**	**-.58**	**.52**	**.41**	**-**
14. Body Gaze Provocation[#]	.01	.11	-.03	-.12	-.03	.04	.10	**.30**	-.11	.15	**.22**	-.17	**.26**
**Women aged > 39 years**													
1. Activewear Wearing	-												
2. Six Month Spend	**.53**	-											
3. Online Browsing	**.30**	**.29**	-										
4. Brand Following	**.40**	**.48**	**.26**	-									
5. Fitness Hours p/week	**.52**	**.43**	**.27**	**.44**	-								
6. Media Pressure	**.29**	**.25**	.03	.06	.05	-							
7. Thin/Low Fat Aspirations	**.24**	.19	.08	.05	.13	**.58**	-						
8. Muscular/Athletic Aspirations	**.37**	**.47**	**.23**	**.29**	**.21**	**.40**	**.54**	-					
9. Self-Esteem	-.01	-.02	-.06	.15	.04	**-.43**	**-.36**	-.08	-				
10. Body Appreciation	.03	.09	-.02	.17	.09	**-.41**	**-.31**	.10	**.76**	-			
11. Appearance Comparisons	**.27**	**.22**	.16	.09	.08	**.61**	**.54**	**.40**	**-.44**	**-.38**	-		
12. Surveillance	**.30**	**.24**	.13	.15	.13	**.53**	**.46**	**.25**	**-.41**	**-.41**	**.65**	-	
13. Shame	.17	**.21**	.13	-.01	.06	**.56**	**.51**	**.30**	**-.64**	**-.60**	**.58**	**.58**	**-**
14. Body Gaze Provocation[#]	.08	**.21**	.12	-.03	-.02	**.21**	**.28**	**.57**	-.12	.06	**.40**	.15	**.20**

Bootstrapping used to estimate all p values. Significant correlations ($p < .01$) are reported in bold. [#] $N = 425$, *$N = 360$

Table S2. *Zero-Order* Intercorrelations between Activewear Engagement Markers and Potential Positive and Negative Psychosocial and Body Image Factors, *Separated by Body Mass Index Groups in the Community Sample.*

	1.	2.	3.	4.	5.	6.	7.	8.	9.	10.	11.	12.	13.
Women < 25 BMI (n = 151)													
1. Activewear Wearing	-												
2. Six Months Spend	**.39**	-											
3. Online Browsing	**.33**	**.68**	-										
4. Brand Following	**.42**	**.40**	**.29**	-									
5. Fitness Hours p/week	**.40**	.19	.19	**.40**	-								
6. Media Pressure	**.31**	.14	.14	.20	.14	-							
7. Thin/Low Fat Aspirations	**.33**	**.34**	**.24**	.21	.11	**.58**	-						
8. Muscular/Athletic Aspirations	**.37**	**.36**	**.25**	**.35**	**.21**	**.47**	**.57**	-					
9. Self-Esteem	.03	-.17	-.08	-.08	.13	**-.30**	**-.38**	-.18	-				
10. Body Appreciation	-.01	-.10	-.01	-.01	.09	-.19	-.16	.11	**.72**	-			
11. Appearance Comparisons	.17	**.25**	.17	.19	.08	**.55**	**.47**	**.37**	**-.38**	**-.31**	-		
12. Surveillance	**.33**	**.28**	.16	**.33**	.19	**.51**	**.48**	**.20**	**-.40**	**-.43**	**.60**	-	
13. Shame	.12	**.27**	**.23**	.15	.02	**.39**	**.48**	**.40**	**-.59**	**-.50**	**.51**	**.46**	**-**
14. Body Gaze Provocation[#]	-.01	.17	.11	-.03	-.04	**.24**	**.28**	**.45**	**-.20**	.06	**.40**	.05	**.31**
Women > 24 BMI (n = 178)													
1. Activewear Wearing	-												
2. Six Month Spend	**.41**	-											
3. Online Browsing	**.29**	**.23**	-										
4. Brand Following	**.46**	**.56**	**.25**	-									
5. Fitness Hours p/week	**.48**	**.44**	**.23**	**.34**	-								
6. Media Pressure	**.34**	.10	.06	.14	.16	-							
7. Thin/Low Fat Aspirations	.16	.08	.08	.04	**.19**	**.54**	-						
8. Muscular/Athletic Aspirations	**.49**	**.41**	**.29**	**.36**	**.29**	**.38**	**.39**	-					
9. Self-Esteem	.02	-.03	-.04	.08	-.04	**-.41**	**-.38**	-.01	-				
10. Body Appreciation	.08	.17	.01	.08	.11	**-.37**	**-.34**	.14	**.75**	-			
11. Appearance Comparisons	**.26**	.12	.16	.11	.16	**.65**	**.60**	**.39**	**-.49**	**-.39**	-		
12. Surveillance	**.28**	.03	.11	.14	.08	**.63**	**.53**	**.27**	**-.41**	**-.41**	**.66**	-	
13. Shame	.18	.02	.03	-.04	.09	**.63**	**.55**	**.22**	**-.62**	**-.54**	**.61**	**.58**	**-**
14. Body Gaze Provocation[#]	.13	.13	.08	-.08	.02	.07	**.22**	**.43**	-.06	.11	**.24**	.01	**.21**

Bootstrapping used to estimate all p values. Significant correlations ($p < .01$) are reported in bold. BMI = Body Mass Index. $^{\#}N = 425$, $^{*}N = 360$
